# Reply to Scheper and de Boer's comment on “Duration of rifampin therapy is a key determinant of improved outcomes in early-onset acute prosthetic joint infection due to Staphylococcus treated with a debridement, antibiotics and implant retention (DAIR): a retrospective multicenter study in France” by Becker et al. (2020) 

**DOI:** 10.5194/jbji-6-19-2020

**Published:** 2020-07-22

**Authors:** Agathe Becker, Louis Kreitmann, Tristan Ferry

**Affiliations:** 1 Service des Maladies Infectieuses et Tropicales, Centre hospitalier universitaire (CHU) de la Croix Rousse, Hospices Civils de Lyon (HCL), Lyon, France; 2 Centre de Référence des Infections Ostéo-Articulaires Complexes (CRIOAc) de Lyon, Lyon, France; 3 Faculté de Médecine Lyon-Est, Université Claude Bernard Lyon 1, Lyon, France; 4 Inserm U1111, Centre international de recherche en Infectiologie (CIRI), Université Claude-Bernard Lyon 1, Lyon, France

Dear editor, We thank Henk Scheper and Mark G. J. de Boer for their valuable comments on our recent paper published in the journal (Becker et al., 2020). The authors raise concerns about several biases that could distort the association between prolonged rifampin therapy and improved outcomes in patients with acute staphylococcal prosthetic joint infection treated with debridement, antibiotics and implant retention (DAIR). We totally agree with their comments, and we have tried to address them. First of all, the impact of rifampin is complex to evaluate, as a delay could be observed between DAIR and the rifampin initiation, the population who receives it could differ from the population who does not, and finally among patients who receive it, its duration could be variable. Concerning the different potential biases, survival bias might arise because patients with early failures are more likely not to “survive” enough to be started on rifampin, thus inflating the proportion of “non-rifampin” patients in the “failure” group. As the median time from DAIR surgery to failure was 16 d, we compared patients receiving rifampin among those with early (<16 d, n=11) and late (≥16 d, n=14) failure and found respectively that 7/11 (64 %) and 10/14 (71 %) had received rifampin (p>0.99 by Fisher's exact test). Then, to address confounding by indication, we compared variables potentially associated with “failure” according to treatment. We found that patients with rifampin (n=58) were younger (71 [61–80] vs. 75 [66–86] years, p=0.046), but the majority of them are >70 years in both groups, and no differences were noticed concerning the prevalence of comorbidities. Even though we used multivariate modelling to control for this bias, we acknowledge that interaction could be searched between treatment and patient characteristics related to practice guidelines. Then, we agree that excluding patients with failure while still on rifampin underestimates the proportion of “rifampin” patients in the “failure” group. However, our aim was to compare the duration of rifampin treatment between patients with failure and success of DAIR, among the patients receiving rifampin (n=58). As suggested by a reviewer, removing patients failing while on rifampin (n=7) limits the bias whereby the proportion of patients with a short duration of rifampin is inflated in the “failure” group (because in those cases rifampin is stopped because failure occurs, and not the opposite). In fact, when considering the whole population (n=79), we still found by multivariate logistic regression that duration of rifampin was independently associated with DAIR failure (OR =0.948, IC95 % =0.893–0.985, p=0.027 for each day of treatment). To limit potential biases evaluating the impact of rifampin, the use of a propensity score could be proposed, but the sample size was here too limited for this approach (D'Agostino, 1998). Finally, the analysis could be treated as a multi-state question where a patient goes from an initial state and could transit to a failure state directly or not, and on which rifampin treatment could influence the transition rate (Putter et al., 2007). Indeed, an event-specific model including rifampin as a time-dependent variable would be a valuable approach to estimate rifampin treatment duration on failure rate.

## Data Availability

Data are not publicly available, as unpublished research on the data set is still ongoing.
